# Abnormality of Resting-State Functional Connectivity in Major Depressive Disorder: A Study With Whole-Head Near-Infrared Spectroscopy

**DOI:** 10.3389/fpsyt.2021.664859

**Published:** 2021-04-29

**Authors:** Eisuke Sakakibara, Yoshihiro Satomura, Jun Matsuoka, Shinsuke Koike, Naohiro Okada, Hanako Sakurada, Mika Yamagishi, Norito Kawakami, Kiyoto Kasai

**Affiliations:** ^1^Department of Neuropsychiatry, Graduate School of Medicine, The University of Tokyo, Tokyo, Japan; ^2^International Research Center for Neurointelligence (IRCN), The University of Tokyo Institutes for Advanced Study (UTIAS), The University of Tokyo, Tokyo, Japan; ^3^University of Tokyo Institute for Diversity & Adaptation of Human Mind (UTIDAHM), Tokyo, Japan; ^4^UTokyo Center for Integrative Science of Human Behavior (CiSHuB), Graduate School of Art and Sciences, The University of Tokyo, Tokyo, Japan; ^5^Center for Evolutionary Cognitive Sciences, Graduate School of Arts and Sciences, The University of Tokyo, Tokyo, Japan; ^6^Department of Mental Health, Graduate School of Medicine, The University of Tokyo, Tokyo, Japan

**Keywords:** major depressive disorder, resting-state functional connectivity, near-infrared spectroscopy, cognitive control network, partial correlation analysis

## Abstract

Near-infrared spectroscopy (NIRS) is a functional neuroimaging modality that has advantages in clinical usage. Previous functional magnetic resonance imaging (fMRI) studies have found that the resting-state functional connectivity (RSFC) of the default mode network (DMN) is increased, while the RSFC of the cognitive control network (CCN) is reduced in patients with major depressive disorder (MDD) compared with healthy controls. This study tested whether the NIRS-based RSFC measurements can detect the abnormalities in RSFC that have been associated with MDD in previous fMRI studies. We measured 8 min of resting-state brain activity in 34 individuals with MDD and 78 age- and gender-matched healthy controls using a whole-head NIRS system. We applied a previously established partial correlation analysis for estimating RSFCs between the 17 cortical regions. We found that MDD patients had a lower RSFC between the left dorsolateral prefrontal cortex and the parietal lobe that comprise the CCN, and a higher RSFC between the right orbitofrontal cortex and ventrolateral prefrontal cortex, compared to those in healthy controls. The RSFC strength of the left CCN was negatively correlated with the severity of depressive symptoms and the dose of antipsychotic medication and positively correlated with the level of social functioning. The results of this study suggest that NIRS-based measurements of RSFCs have potential clinical applications.

## Introduction

Near-infrared spectroscopy (NIRS) is a non-invasive and convenient neuroimaging technique that measures changes in the cortical blood oxygenation associated with neural activity. This has been widely used to study the cerebral function of various neuropsychiatric disorders (1). Among others, a meta-analysis of 14 studies found a significant reduction in the executive task-related elevation of blood oxygenation in individuals with MDD, even after their depressive symptoms had remitted (2). The differences in blood oxygenation patterns during executive tasks in MDD, bipolar disorder, and schizophrenia have led to their clinical application for the differential diagnosis of psychiatric disorders in patients presenting with depressive symptoms (3).

The brain exhibits neural activity even at rest. Since the discovery of synchronous neural oscillatory activity between functionally-related brain regions, there has been a surge of research on resting-state functional connectivity (RSFC) (4). RSFC may provide clues on the mechanisms of functional integration in the brain and the neural substrate of neuropsychiatric disorders (5, 6).

Most previous studies on neuropsychiatric disorders, focusing on RSFC, have used functional magnetic resonance imaging (fMRI). Emotion regulation circuits comprising the cortex and limbic system were found dysfunctional in MDD (7). However, this dysfunction is not detected in NIRS because NIRS does not measure activities in deep brain regions. In contrast, recent studies have reported that the RSFC of the default mode network (DMN), comprising the medial prefrontal and posterior cingulate cortices, and bilateral parietal lobes, is increased, while the RSFC of the cognitive control network (CCN), comprising the frontal and parietal lobes, is reduced in patients with MDD compared with healthy controls (8, 9).

We have established the method for measuring RSFC between 17 cortical regions from NIRS blood oxygenation signals using partial correlation analysis, which has been demonstrated to reduce the influence of extracerebral blood flow (10). NIRS has certain advantages over fMRI in the clinical application of the RSFC measurement. First, NIRS has a higher temporal resolution (10 Hz) than fMRI (about 0.5 Hz); thus, it avoids the aliasing of cardiac or respiratory signals. Second, it is less expensive, convenient to set up, and more tolerant to motion artifacts than fMRI. Third, it can measure the cortical activity while the participant is sitting in a natural position in any ordinary room (10). The purpose of this study was to test whether the previously established NIRS-based RSFC measurements can detect the abnormalities in RSFCs of MDD patients, as reported in previous fMRI studies. Because these reports included the RSFC between superficial cerebral cortices, we hypothesized that the hyper- and hypoconnectivity of the DMN and CCN, respectively, in MDD patients can be detected by the NIRS-based RSFC measurement.

## Methods

### Ethics

The study was performed following the Declaration of Helsinki and all participants provided written informed consent. This study was approved by The Research Ethics Committee of the Faculty of Medicine of the University of Tokyo (Approval no. 630, 3202, 3361). Written informed consent was obtained from the individual for the publication of any potentially identifiable images or data included in this article.

### Participants

For the MDD group, a total of 71 individuals with depressive symptoms were recruited-−25 were admitted to the Department of Neuropsychiatry at the University of Tokyo Hospital between April 2015 and February 2020 for the assessment or treatment of depressive symptoms, and the remaining 46, who participated in the longitudinal study on the brain functional bases and changes in depressive psychiatric disorders, underwent RSFC measurements and clinical assessments (11). The inclusion criteria were current or past MDD defined by the Diagnostic and Statistical Manual Mental Disorders, 4th edition, through a structured clinical interview (12). The exclusion criteria were comorbid dysthymic disorder or substance-induced mood disorder, a history of coarse brain organic disease or epilepsy, alcohol dependence, use of illicit drugs at the time of the study, a childhood diagnosis of mental retardation or developmental disability, a history of loss of consciousness for more than 5 min, and an active physical illness that could present with psychiatric symptoms. Consequently, 51 individuals with current or past MDD were selected.

Of these 51 individuals, fifteen individuals who fell asleep during the NIRS measurements or reported sleepiness grades of 4 or more on the Stanford sleepiness scale (SSS, 0 [feeling active] to 7 [sleep onset]) were excluded from the analysis (13). Additionally, an automatic artifact detection algorithm was applied to the NIRS data, and two participants, for whom the cerebral region-averaged signals could not be estimated owing to excessive artifacts, were excluded (10). Following all the exclusions, we classified the remaining 34 participants as the MDD group. There were no significant differences in age, sex, and years of education between the 34 included and 37 excluded individuals in this study (*p* > 0.15).

For the healthy control (HC) group, 176 participants were recruited from the original pool of participants of the Japanese Study of Stratification, Health, Income, and Neighborhood (J-SHINE) survey, a population-representative survey in the metropolitan region of Tokyo (14). Of these 176 participants, 61 participants with psychiatric disorders detected using the Japanese version of the Composite International Diagnostic Interview or current use of psychotropic drugs were excluded (15, 16). Of these 115 participants, 35 participants who fell asleep during the measurements or were reported with 4 or more points on the SSS, and two participants whose cerebral region-averaged signals could not be estimated owing to excessive artifacts were excluded. The remaining 78 individuals were classified as the HC group.

The rate of participants who were excluded due to sleepiness or excessive artifacts was not significantly different between HC and MDD group (37/115 vs. 17/51, *p* = 0.89).

For both HC and MDD groups, the participants' age, sex, and years of education were assessed. Each participant's estimated (premorbid) intelligence quotient (eIQ) was assessed using the 25-item Japanese adult reading test (17, 18). Besides, we used the Center for Epidemiologic Studies depression scale (CESD) to evaluate the severity of the subjective depressive symptoms (19).

For the MDD group, a psychiatrist or clinical psychologist assessed the severity of the objective depressive symptoms using the 17-item Hamilton rating scale for depression (HAMD) (20). Social functioning was assessed using the global assessment of functioning (GAF) (21). We also calculated the imipramine equivalent for antidepressants, diazepam equivalent for anxiolytics, and chlorpromazine equivalent for antipsychotics, at the time of NIRS measurement (22).

### NIRS Measurement

**Figure 1:**
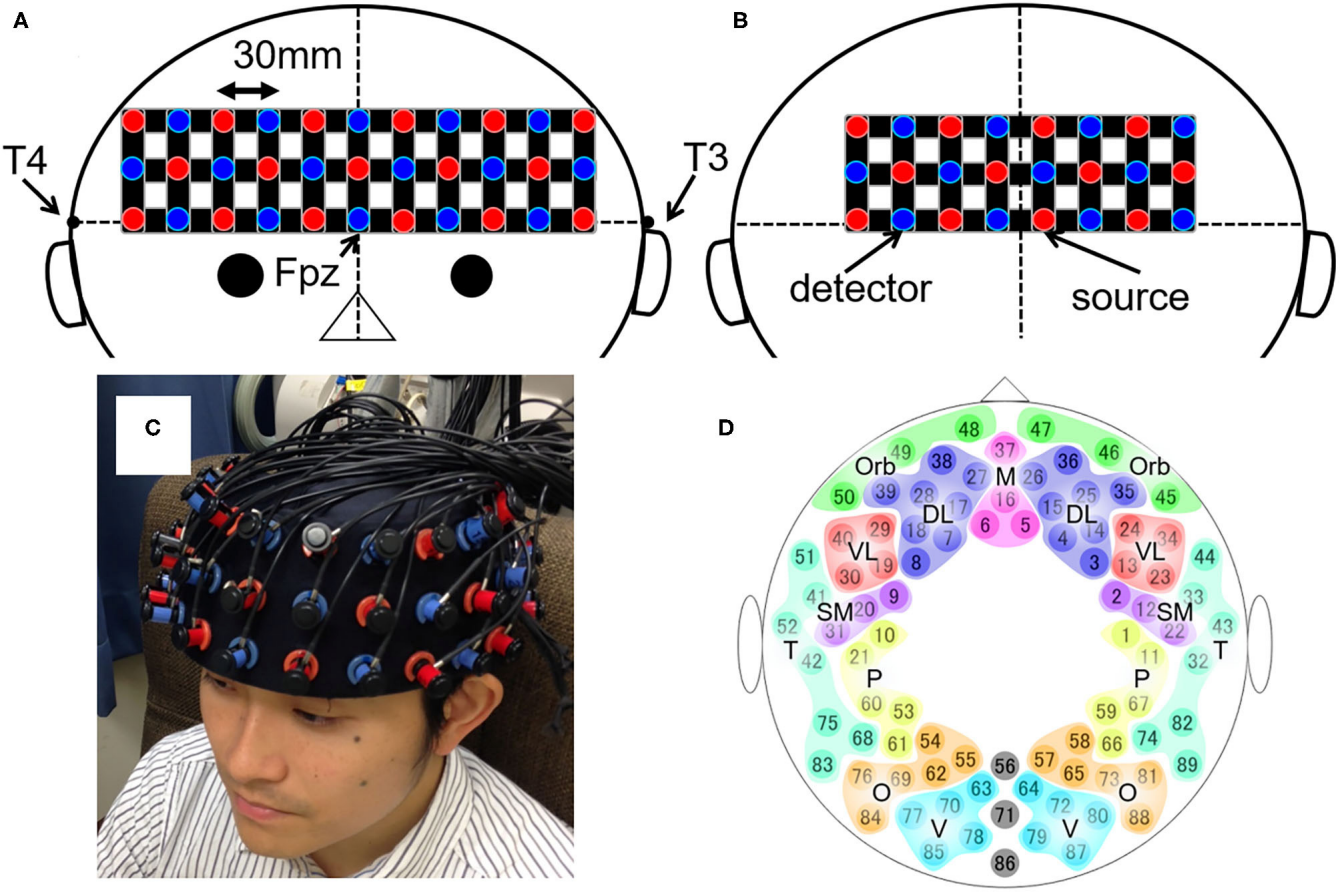
The layout of the whole-head NIRS system and the 89 measuring points. **(A,B)** Schematic illustration of **(A)** the anterior and **(B)** the posterior shells of the whole-head NIRS arrangement. The red and blue circles depict the source and detector probes, respectively. The black horizontal line represents the planar surface defined by T3, Fpz, and T4 according to the international 10–20 system for electroencephalography. The black vertical line represents the midline. **(C)** The actual placement of the probes on the head. **(D)** Schematic illustration of the 89 measuring points and their categorization into 17 brain regions. DL, dorsolateral prefrontal cortex; M, medial prefrontal cortex; Orb, orbitofrontal cortex; VL, ventrolateral prefrontal cortex; SM, sensorimotor area; P, parietal lobe; T, temporal lobe; O, occipital lobe; V, visual area.

The measurement protocol was identical to our previous study (10). Participants were seated upright in a quiet room with eyes closed and instructed to stay awake. Changes in the relative concentrations of oxygenated hemoglobin (oxy-Hb) and deoxygenated hemoglobin (deoxy-Hb) were measured for 8 min using a whole-head NIRS arrangement (composed of two ETG-4000 machines from Hitachi, Tokyo, Japan). The system was composed of anterior and posterior thermoplastic shells (Figures 1A,B). The anterior shell contained 17 source and 16 detector probes. The posterior shell contained 12 source and 12 detector probes. The source and detector probes were placed alternately within the shells; this constituted 89 source-detector pairs (henceforth, channels), each distanced by 30 mm. Participants wore the probes such that the lowest probes were located on the planar surface defined by T3, Fpz, and T4, according to the international 10–20 system for electroencephalography (Figure 1C). This probe arrangement allowed the measurement of changes in oxy- and deoxy-Hb from the bilateral frontal, temporal, parietal, and occipital regions of the cortical surface (Figure 1D).

### Data Preparation

Data were prepared using MATLAB R2020b (MathWorks, Inc., MA, USA). We applied the procedure previously established to estimate RSFCs between the predetermined 17 cerebral regions, for each oxy- and deoxy-Hb signal (10). Recapitulating the procedure, the signals containing significant artifacts were excluded using an automated algorithm. Then, the region-averaged signals for the 17 cerebral regions were calculated from the oxy- and deoxy-Hb signals acquired from 89 channels (Figure 1D). We used a Butterworth band-pass filter to extract the 0.009–0.08 Hz signal component. Finally, we defined the partial correlation coefficient of the signals from two given brain regions as the index of RSFC between the regions.

### Statistical Analysis

Statistical analysis was performed using MATLAB R2020b and IBM SPSS Statistics 27 (IBM Inc., New York, USA). We examined whether there were significant differences in age, sex ratio, years of education, eIQ, and CESD scores between the MDD and HC groups. A chi-square test was used for the sex-ratio analysis, and unpaired two-sample *t*-tests were used for analyzing the other factors.

Statistical tests for the RSFC indices were performed by transforming the partial correlation coefficients into z-values using Fisher's z-transformation. Of the 136 brain region pairs, those with partial correlation coefficients of 0.1 or higher in the HC group were regarded as connected. For each connected pair, the difference in RSFCs between the MDD and HC groups was tested using the two-samples unpaired *t*-test.

Because this was a preliminary study, the significance level was set at 5% without any false discovery rate (FDR) correction. However, we also examined the significance of the differences after adjusting for FDR (23).

Besides, we tested the correlations between RSFCs and various clinical variables, including the HAMD, CESD, and GAF scores, and the amounts of imipramine equivalents of antidepressants, diazepam equivalents of anxiolytics, and chlorpromazine equivalents of antipsychotics taken by the participants, for each connected region-pair in the MDD group. The normality of the clinical variables was tested using the Shapiro-Wilk test; Pearson's and Spearman's rank correlation coefficients were obtained when the normality was not rejected and was rejected, respectively.

## Results

### Demographic and Clinical Features of the MDD and HC Groups

The demographic and clinical features of the participants (34 and 78 in the MDD and HC groups, respectively) are shown in Table 1. There was no significant difference in age, sex ratio, years of education, and SSS between the MDD and HC groups (*p* > 0.11). In contrast, eIQ (112.0 vs. 107.5; Cohen's *d* = 0.501, 95% confidence intervals (CI) [0.092 0.908], *p* = 0.02) and CESD (20.0 vs. 8.1; Cohen's *d* = 1.413, 95% CI [0.937 1.882], *p* < 0.001) were significantly higher in the MDD than in the HC group. HAMD and GAF scores and medications used by the MDD participants are shown in Table 1. Twelve MDD participants were taking mood stabilizers (five used lithium, three lamotrigine, and eight valproate, with overlap).

**Table 1:** Demographic characteristics of participants.

	**MDD**		**HC**		**Statistical values**
	**Mean**	**SD**	**Mean**	**SD**	
Sex (m/f)	17/17		36/42		$\chi_{\left(1\right)}^{2}$ = 0.141; *p* = 0.71
Age (years)	37.4	9.9	37.3	7.2	*t*_(110)_ = 0.093; *p* = 0.93
Education (years)	15.7	2.2	15.1	2	*t*_(101)_ = 1.357; *p* = 0.18
eIQ	112	8	107.5	9.3	*t*_(110)_ = 2.440; *p* = 0.02
CESD	20	14	8.1	5.2	$t_{\left(29.7\right)}^{*}$= 4.391; *p* < 0.001
SSS	2.74	0.51	2.54	0.73	$t_{\left(88.3\right)}^{*}$= 1.631; *p* = 0.11
HAMD	7.12	6.29			
GAF	57.4	16.4			
IMP	96.5	151.9			
CP	48.1	105.1			
DZP	4.9	8.7			

*MDD, major depressive disorder; HC, healthy control; SD, standard deviation; m: male; f, female; eIQ, estimated (premorbid) intelligence quotient; SSS, Stanford sleepiness scale; HAMD, 17-item Hamilton rating scale for depression; CESD, Center for Epidemiologic Studies depression scale; GAF, global assessment of functioning; IMP, imipramine equivalent of antidepressant dosage; CP, chlorpromazine equivalent for antipsychotic dosage; DZP, diazepam equivalent for anxiolytic dosage*.

*The degrees of freedom were adjusted for CESD and SSS because the equality of variances between the groups was rejected.

### Differences in RSFC Patterns Between the MDD and HC Groups

**Figure 2:**
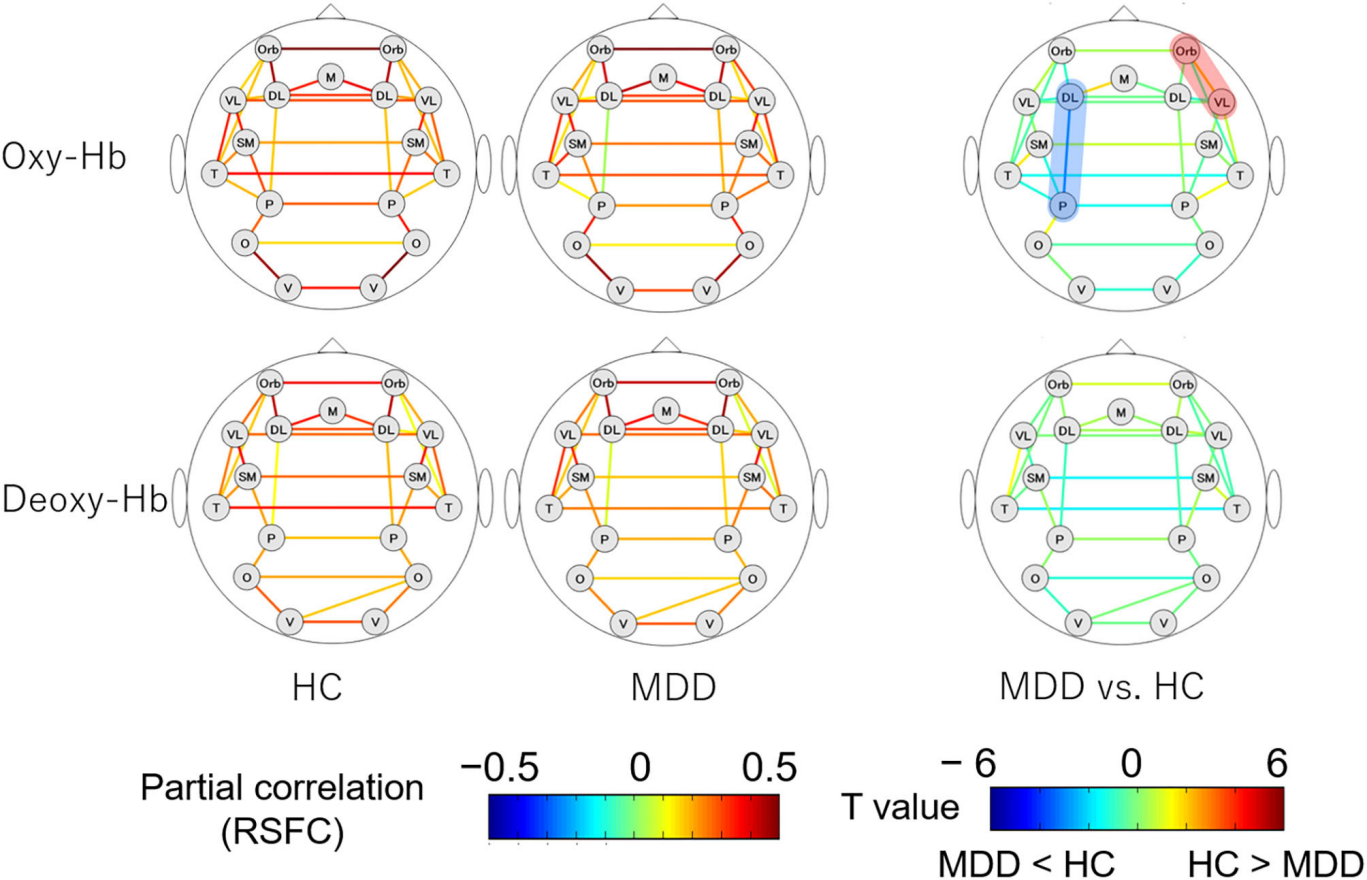
RSFC patterns of the MDD and HC groups. The upper and lower rows show the RSFC patterns calculated from oxy- and deoxy-Hb signals, respectively. The figures on the left illustrate the RSFC patterns of the HC group and the figures in the middle illustrate the RSFC patterns of the MDD group. The figures on the right show the comparison of the strengths of RSFCs between the MDD and HC groups. The brain region pairs, where the strength of RSFC was significantly different between the groups are highlighted in red (MDD > HC) or blue (HC > MDD). MDD, major depressive disorder; RSFC, resting-state functional connectivity; HC, healthy control; oxy-Hb, oxygenated hemoglobin; deoxy-Hb, deoxygenated hemoglobin; DL, dorsolateral prefrontal cortex; M, medial prefrontal cortex; Orb, orbitofrontal cortex; VL, ventrolateral prefrontal cortex; SM, sensorimotor area; P, parietal lobe; T, temporal lobe; O, occipital lobe; V, visual area.

The RSFC patterns and *t*-tests results of the MDD and HC groups are shown in Figure 2. The MDD and HC groups had similar RSFC patterns. For oxy-Hb, the RSFC between the left dorsolateral prefrontal cortex (DLPFC) and parietal lobe was significantly lower in the MDD than in the HC group (*z* = 0.038 vs. 0.171; Cohen's *d* = 0.583, 95% CI [0.172 0.992], *p* = 0.005). The RSFC between the right orbitofrontal cortex (OFC) and ventrolateral prefrontal cortex (VLPFC) was significantly higher in the MDD than in the HC group (*z* = 0.315 vs. 0.202; Cohen's *d* = 0.464, 95% CI [0.055 0.870], *p* = 0.03). For deoxy-Hb, there were no region pairs with significant differences in RSFCs between the MDD and HC groups. In both oxy- and deoxy-Hb, the RSFCs between the bilateral parietal lobes did not differ significantly between the two groups. There was no brain region pair in which the difference in RSFCs between the groups was significant after FDR correction.

### The Correlation Between RSFCs and Clinical Variables in the MDD Group

**Figure 3:**
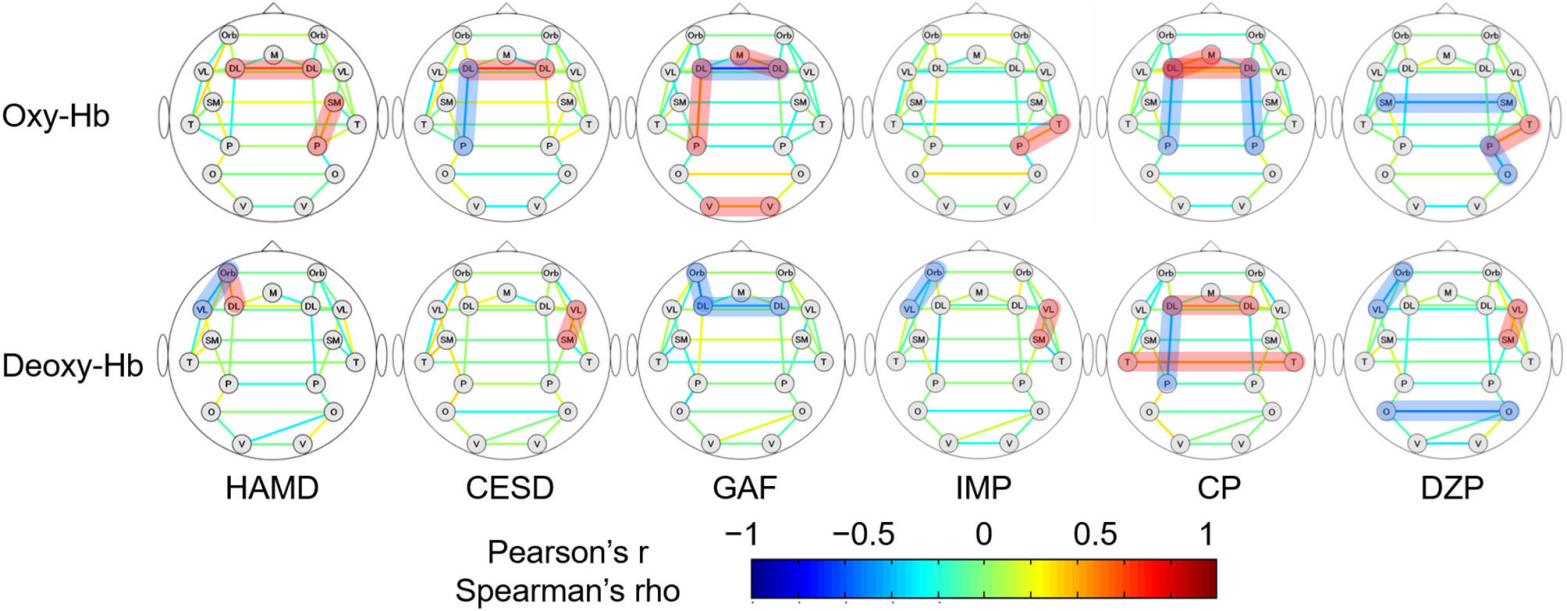
The association between the strengths of RSFCs and clinical variables in MDD patients. The upper and lower rows show the RSFC patterns calculated from oxy- and deoxy-Hb signals, respectively. Pearson's correlation coefficients are depicted for CESD and GAF, while Spearman's rank correlation coefficients are depicted for the rest. The brain region pairs, where the correlation between the strengths of RSFCs and the clinical variables were significantly different between the groups are highlighted in red (positive correlation) or blue (negative correlation). HAMD, 17-item Hamilton rating scale for depression; CESD, Center for Epidemiologic Studies depression scale; GAF, global assessment of functioning; IMP, imipramine equivalent of antidepressant dosage; CP, chlorpromazine equivalent for antipsychotic dosage; DZP, diazepam equivalent for anxiolytic dosage; DL, dorsolateral prefrontal cortex; M, medial prefrontal cortex; Orb, orbitofrontal cortex; VL, ventrolateral prefrontal cortex; SM, sensorimotor area; P, parietal lobe; T, temporal lobe; O, occipital lobe; V, visual area.

The correlations between RSFCs obtained from oxy- and deoxy-Hb signals and various clinical variables in 34 MDD patients are shown in Figure 3. Pearson's correlation coefficients were calculated for CESD and GAF scores (normally distributed); Spearman's rank correlation coefficients were calculated for HAMD, antidepressant, antipsychotic, and anxiolytic doses (not normally distributed).

For oxy-Hb, the RSFC between the left DLPFC and parietal lobe was negatively correlated with CESD (*r* = −0.405, 95% CI [−0.676 −0.037], *p* = 0.03) and the antipsychotic dose (*ρ* = −0.400, 95% CI [−0.658 −0.058], *p* = 0.02), and positively correlated with GAF (*r* = 0.452, 95% CI [0.110 0.699], *p* = 0.01). The RSFC between the left and right DLPFCs was positively correlated with HAMD (*ρ* = 0.541, 95% CI [0.225 0.754], *p* < 0.001), CESD (*r* = 0.619, 95% CI [0.320 0.806], *p* < 0.001), and antipsychotic dose (*ρ* = 0.473, 95% CI [0.141 0.709], *p* = 0.005), and negatively correlated with GAF (*r* = −0.720, 95% CI [−0.858 −0.486], *p* < 0.001). Other positive correlations were found between HAMD and the RSFCs of the right sensorimotor cortex and the parietal lobe (*ρ* = 0.356, 95% CI [0.009 0.626], *p* = 0.04), GAF and the RSFCs of the left and right visual area (*r* = 0.374, 95% CI [0.016 0.647], *p* = 0.04), and GAF and the RSFCs of the right DLPFC and the medial prefrontal cortex (*r* = 0.407, 95% CI [0.054 0.669], *p* = 0.03). Correlation trends were inconsistent between the medications and the RSFCs of other brain region pairs. Correlations between the three clinical variables (HAMD, CESD, and GAF) and RSFCs of the left and right DLPFCs were significant following FDR correction.

For deoxy-Hb, HAMD correlated negatively with the RSFC between the left OFC and VLPFC (*ρ* = −0.350, 95% CI [−0.622 −0.002], *p* = 0.04) and positively with that between the left OFC and DLPFC (*ρ* = 0.371, 95% CI [0.026 0.637], *p* = 0.03). CESD correlated positively with the RSFC between the right VLPFC and sensorimotor area (*r* = 0.410, 95% CI [0.043 0.679], *p* = 0.03). GAF correlated negatively with the RSFC between the left and right DLPFCs (*r* = −0.380, 95% CI [−0.651 −0.023], *p* = 0.04) and that between the left OFC and DLPFC (*r* = −0.489, 95% CI [−0.722 −0.157], *p* = 0.006). The RSFC between the left DLPFC and parietal lobe correlated only with the antipsychotic dose (*ρ* = −0.404, 95% CI [−0.661 −0.062], *p* = 0.02). The correlation trends between medications (doses) and RSFCs of other brain region pairs were inconsistent. However, there was no brain region pair in which the RSFC significantly correlated with the clinical variables following FDR correction.

### Analyses of Group Differences in RSFCs Between the Left DLPFC and Parietal Lobe

The RSFC between the left DLPFC and parietal lobe, which was significantly different between the MDD and HC groups, was correlated with the CESD, GAF, and antipsychotic medication dosage of the MDD group. Since GAF was not obtained for the HC group, we performed an analysis of covariance with the RSFC estimated from the oxy-Hb signals of the left DLPFC and parietal lobe as the dependent variable, the group (i.e., MDD or HC) as the fixed factor, and the CESD and dosage of antipsychotic medication as covariates. The results showed that after controlling for CESD and antipsychotic medication, the group differences in RSFCs between the left DLPFC and parietal lobe ceased to be significant, leaving the trend to be lower in the MDD than in the HC group [*F*_(1,102)_ = 2.568, partial η^2^ = 0.25, *p* = 0.11].

## Discussion

### Summary of Findings

This is the first study that utilized a whole-head NIRS system for elucidating RSFC abnormalities in MDD patients, applying the partial correlation analysis established previously. We found that the MDD group had a lower RSFC between the left DLPFC and parietal lobe and higher RSFC between the right OFC and VLPFC, compared to the HC group.

Furthermore, the RSFCs were correlated with various clinical variables in the MDD group. Particularly, the RSFC between the left DLPFC and parietal lobe, which was significantly lower in the MDD than in the HC group, was negatively correlated with the CESD and antipsychotic dose and was positively correlated with GAF. Besides, the RSFC between the left and right DLPFCs was positively correlated with the HAMD, CESD, and antipsychotic dose and negatively correlated with GAF.

### Characteristic RSFC Patterns in MDD Patients

Mood disorders are psychiatric disorders characterized by mood dysregulation. Functional imaging studies in MDD have long focused on impairments in emotion regulation circuits, consisting of the limbic system and its connectivity with cortical regions (7, 24, 25). Recently, MDD has been associated with a variety of cognitive dysfunctions (26, 27), including, reduced psychomotor speed, memory, cognitive flexibility, and word fluency, as shown by a meta-analysis (28). Some of these cognitive impairments may persist even after symptomatic remission (29).

Other studies suggest that MDD patients have a reduced RSFC in the CCN and a stronger RSFC in the DMN, compared to those in healthy individuals (8, 9). A strong RSFC in the DMN causes the self-referential process of rumination, where negative thoughts, such as regret, self-loathing, hopelessness, or worry, repetitively occur in the individual (30).

In this study, the RSFC between the bilateral parietal lobes, which may reflect the DMN connectivity, did not differ between the two groups. In contrast, the RSFC between the left DLPFC and parietal lobe, which may reflect an impaired cognitive function, was weaker in the MDD than in the HC group.

Besides, the RSFC between the right OFC and VLPFC increased in MDD patients. A recent study on 282 MDD patients revealed that the RSFC between the right inferior frontal gyrus and other brain regions, including the OFC, was higher in MDD patients than in healthy individuals (31). In our study, the inferior frontal gyrus is classified as the VLPFC; therefore, our findings are in line with previous findings. The inferior frontal gyrus and OFC are activated when a subject successfully inhibits natural responses during the stop-signal task (32). Therefore, increased RSFC may be related to decreased motivation and excessive psychomotor inhibition found in MDD patients (31).

RSFC studies using NIRS are scarce. In a study that recruited 28 patients with mood disorders (i.e., bipolar disorder or MDD), the local RSFC among the right inferior frontal gyrus and the RSFC between the bilateral inferior frontal gyrus were significantly lower in the patients than in healthy controls (33). Another study that compared the prefrontal and parietal cortical activities of 49 individuals with late-life MDD and 51 non-depressive individuals during rest and trail making test showed reduced RSFC in the left frontopolar cortical network during the trail making test and increased RSFC in the left CCN at rest (34). In another study, the same research group recruited 60 MDD patients and 24 healthy individuals and discovered that the RSFC in the cortical part of the DMN was lower in MDD patients than in healthy individuals, and rumination was negatively correlated with the strength of RSFC in that region (35).

In the present study, the RSFC in the left CCN was low while that in the prefrontal regions was partially elevated in MDD patients. Although these results were consistent with previous fMRI studies on RSFC, they contradict the results obtained in previous NIRS studies on RSFC. These discrepancies may be attributed to differences in the arrangement of probes, ages of subjects, and the method of estimating the strength of RSFCs. Better consistency with previous fMRI studies could be explained by the usage of partial correlation coefficient as an index of RSFC because it reduces the influence of extracerebral blood flow than Pearson's correlation coefficient used in the previous NIRS studies.

### State Dependence and Pharmacological Influence of RSFC

In our study, the RSFC of the left CCN was negatively correlated with CESD and positively correlated with GAF. Previous studies have examined correlations between various aspects of depressive symptoms and RSFCs (30). A recent meta-analysis, integrating data from 25 publications and 516 MDD patients, found that RSFCs of the CCN and DMN were not correlated with symptom severities (8). In contrast, a study that recruited individuals with subthreshold depressive symptoms reported that CESD scores were negatively correlated with the RSFC between DLPFC and the temporoparietal junction (36). Therefore, the correlation between the RSFC of the left CCN and severity of depressive symptoms in the present study may reflect the fact that this study included many remitters, and the reduced RSFC of the CCN was normalized by the remitters.

In the present study, the RSFC between bilateral DLPFCs was positively correlated with depressive symptoms and negatively correlated with GAF. These results are in agreement with a previous report that stated that depressive symptoms and inter-hemispheric RSFC in the anterior subnetwork of the DMN were negatively correlated (37). In another study, researchers found that the RSFC between what they call “dorsal nexus,” a part of the bilateral dorsomedial prefrontal cortex, and other brain regions, including, other prefrontal regions, the anterior and posterior cingulate cortex, and the precuneus, increased in MDD patients (38). They interpret this as a “hot-wiring” interconnect between DMN, CCN, and affective networks, which incubate various depressive symptoms. Therefore, the results in this study may indirectly reflect an increase in the RSFC between prefrontal regions mediated by the dorsal nexus.

In our study, the antidepressant medication dose did not correlate with the RSFC of the CCN, while the antipsychotic medication dose correlated negatively with the RSFC of the bilateral CCN and positively with the RSFC between the bilateral DLPFCs. In the aforementioned meta-analysis, antidepressants did not correlate with the RSFC of the CCN but normalized the hyperconnectivity in the DMN of MDD patients (9). A study that distinguished the anterior and posterior subnetworks of the DMN, where the posterior DMN subnetwork consisted of the bilateral precuneus and part of the parietal cortex, normalized the hyperconnectivity after antidepressant treatment (39).

Although the effects of antipsychotic medications on depressed patients have not been reported, antipsychotic medications in schizophrenia patients increased RSFCs between the striatum and anterior cingulate gyrus, DLPFC, hippocampus, and anterior insula, and decreased the RSFC between the striatum and parietal lobe, as psychotic symptoms improved. In another study, schizophrenia patients showed increased RSFCs in the right superior temporal gyrus, right medial frontal gyrus, and left superior frontal gyrus, and decreased RSFCs in the right posterior cingulate and precuneus of the DMN, after antipsychotic treatment (40). Besides, the patients exhibited decreased RSFCs in the right cerebellum anterior lobe and left insula in the salience network. The changes in RSFCs reported in these previous studies included brain regions that cannot be measured with NIRS. Furthermore, the previous studies compared the RSFCs before and after medication in the same subjects; this may be difficult to extrapolate for interpreting the variance among different subjects with different medications, as is the case in the present study. The results of this study suggest that psychotropic medications have substantial effects on RSFCs and different kinds of psychotropics have different influences.

### Limitation of the Study

First, the differences in RSFCs between the HC and MDD groups and the correlations between RSFCs and clinical variables in the MDD group were not statistically significant after FDR correction, except for the association between clinical variables and the RSFC between the left and right DLPFCs. In addition, we performed a *post-hoc* sensitivity analysis using G^*^Power 3 (41). If we set α = 0.05 and β = 0.80, the sample size of our study (*n* = 34 and 78) is estimated to have the power to detect the group difference with an effect size of 0.58 or more, a medium size effect. Therefore, smaller effect size differences may be undetected in our study. Furthermore, the importance of assessing the reliability of measurement methods in neuroimage research has been indicated (42). However, there is no data on the reliability of the method used in this study. Therefore, the reproducibility of the results needs to be validated using larger datasets in the future, and the assessment of reliability of the method for estimating the strength of the RSFC used in this study should be clarified.

Second, in this study, the MDD patients were referred to the University of Tokyo Hospital, a tertiary care hospital, for the assessment of depressive symptoms that were refractory to treatment. The high percentage of patients taking antipsychotic medications and mood stabilizers, despite being diagnosed with MDD according to the Diagnostic and Statistical Manual Mental Disorders, indicates that the subjects in this study were not typical MDD patients. The study also included many patients from the longitudinal study, many of whom were already in the remission phase of their depressive symptoms. Besides, this study suggests that antipsychotic medications have various effects on RSFCs. Therefore, the RSFC characteristics and their correlations with clinical variables in the MDD group are likely to be influenced by antipsychotic medications. Therefore, careful consideration is required when the study results are extrapolated to a more typical depressed group, who are non-medicated or solely taking antidepressants.

## Summary

The present study used a whole-head NIRS system to elucidate RSFC patterns characteristic of MDD. Compared with the HC group, MDD patients had a lower RSFC between the left DLPFC and parietal lobe, which may constitute the CCN. The RSFC in the CCN was negatively correlated with the severity of depressive symptoms and dosage of antipsychotic medication and positively correlated with the level of functioning. Therefore, these might reflect an individual's depressive status rather than the trait predisposing to depression. This study confirms that abnormalities in RSFC patterns in major depressive disorders, as identified by previous fMRI studies, can be partially detected by measuring the resting-state brain activities using NIRS. Combining the convenience of measuring the brain activity using NIRS and the ease of performing resting-state measurements (no requirement for cognitive tasks), NIRS-based measurements of RSFCs have potential clinical applications. Therefore, further studies validating the result of our findings and those of the NIRS-based RSFC measurements in other psychiatric disorders are warranted.

## Data Availability Statement

The datasets analyzed during the current study are not publicly available due to ethical considerations in this study but are available from the corresponding author on reasonable request, with the approval of the Research Ethics Committee of the Faculty of Medicine of the University of Tokyo.

## Ethics Statement

The studies involving human participants were reviewed and approved by The Research Ethics Committee of the Faculty of Medicine of the University of Tokyo (Approval No. 630, 3202, 3361). The patients/participants provided their written informed consent to participate in this study.

## Author Contributions

YS, NK, and KK designed the study. Data were collected by ES, YS, JM, MY, HS, and NK. ES analyzed and interpreted the data with intellectual input from YS, JM, SK, NO, HS, MY, and KK. ES wrote the manuscript, which was revised by YS, SK, and KK. All the authors have approved the final manuscript.

## Conflict of Interest

The authors declare that the research was conducted in the absence of any commercial or financial relationships that could be construed as a potential conflict of interest.

## Abbreviations

MDD: major depressive disorder
NIRS: near-infrared spectroscopy
fMRI: functional magnetic resonance imaging
RSFC: resting-state functional connectivity
DMN: default mode network
CCN: cognitive control network
HC: healthy control
eIQ: estimated (premorbid) intelligence quotient
SSS: Stanford sleepiness scale
HAMD: 17-item Hamilton rating scale for depression
CESD: Center for Epidemiologic Studies depression scale
GAF: global assessment of functioning
oxy-Hb: oxygenated hemoglobin
deoxy-Hb: deoxygenated hemoglobin
FDR: false discovery rate
DLPFC: dorsolateral prefrontal cortex
OFC: orbitofrontal cortex
VLPFC: ventrolateral prefrontal cortex.
